# Prediction models for venous thromboembolism in pediatric patients: A protocol for systematic review and critical appraisal

**DOI:** 10.1371/journal.pone.0361300

**Published:** 2026-11-04

**Authors:** Huiqin Liu, Xiaojing Zhou, Zifeng Li, Wei Lu, Qianqian Ding, Zuyang Xi, Jingjing Yang

**Affiliations:** 1 Department of Pediatrics, The First College of Clinical Medical Science, China Three Gorges University, Yichang Central People’s Hospital, Hubei Province, China; 2 Department of Traditional Chinese Medicine, The First College of Clinical Medical Science, China Three Gorges University, Yichang Central People’s Hospital, Hubei Province, China; 3 Department of Neonatal Intensive Care Unit, The First College of Clinical Medical Science, China Three Gorges University, Yichang Central People’s Hospital, Hubei Province, China; 4 Department of Nursing, The First College of Clinical Medical Science, China Three Gorges University, Yichang Central People’s Hospital, Hubei Province, China; 5 Department of Pediatric Outpatient, The First College of Clinical Medical Science, China Three Gorges University, Yichang Central People’s Hospital, Hubei Province, China; Celal Bayar University: Manisa Celal Bayar Universitesi, TÜRKIYE

## Abstract

Venous thromboembolism (VTE) in children is a severe disease with high mortality rates. It is also gradually recognized as a major source of morbidity in hospitalized children, leading to increased length of hospital stay, higher mortality, and increased costs. The majority of pediatric subjects with VTE present inherited or acquired risk factors,such as the use of a central venous catheter (CVC), which accounts for more than more than 90% of neonatal VTE and 50% of pediatric VTE. Clinical studies on predictive models for VTE in children have shown inconsistent results. This systematic review aims to evaluate published prediction models for pediatric VTE, analyzing their characteristics, predictors, efficacy, and the methodological quality and applicability. The protocol has been prepared using the Preferred Items for Systematic Review and Meta-analysis Protocols (PRISMA-P) guidelines. The systematic review protocol for VTE prediction in pediatric patients has been registered on PROSPERO (PROSPERO 2025CRD420251231316. Available from https://www.crd.york.ac.uk/PROSPERO/vi-ew/CRD420251231316.) A comprehensive search across five major databases (PubMed, Web of Science, Cochrane Library, CNKI, and Wanfang) will be conducted for studies on prediction models of VTE among pediatric patients. Two researchers will independently screen literature, extract data, and evaluate the bias risk and applicability of included studies using prediction modelling study tools. This systematic review will provide critical insights into the efficacy and quality of reporting of the VTE model in pediatric patients. This will guide clinical physicians in selecting the most appropriate VTE prediction model and inform future research endeavors in pediatric VTE prediction.

## Introduction

Venous thromboembolism (VTE) is a venous return disorder in which blood clots abnormally in the veins, causing complete or incomplete blockage of the blood vessels, including deep vein thrombosis (DVT) and pulmonary embolism (PE) [1]. Although pediatric venous thromboembolism (VTE) is a rare condition, the incidence of VTE among all hospitalized pediatric patients keeps rising, and an increasing number of health care professionals are involved in its management due to this rise over the past two decades [2]. VTE in children is a severe disease with high mortality rates and can cause acute and chronic related complications [3]. It is also gradually recognized as a major source of morbidity in hospitalized children, leading to increased length of hospital stay, higher mortality, and increased costs [4–7]. However, the epidemiological burden of VTE in pediatric patients remains insufficiently studied on a global scale [8], no standardized mechanism exists to estimate the risk of VTE among individual patients [9], and there is a lack of consensus on anticoagulant prophylaxis strategies [10].

VTE in children is increasingly observed and diagnosed due to the greater understanding of this disease, the increased survival rate in patients with chronic conditions, and the more frequent use of catheters and interventional procedures, as well as improved diagnostic techniques [11,12]. It was reported that the incidence of VTE in hospitalized children in China had increased from 0.024% in 2013 to 0.055% in 2016 [13], putting heavy financial burden on both the patients and the whole society. The total annual health-care costs associated with venous thromboembolism are estimated to range between €1.5–3.3 billion in Europe and $7–10 billion in the USA [14,15].

The mainstay of treatment and secondary prevention of VTE is anticoagulation therapy. The major advancement in management for VTE in adult in the past decade has been the introduction of direct oral anticoagulants [16]. In light of heterogeneous recommendations and the lack of data on the optimal interventional treatment, the most suitable treatment option for pediatric patients remains uncertain [8], with very little specific research in children. Much of the evidence for treatment is inferred from adult practice, despite the major differences between adults and children in the epidemiology and pathophysiology of thrombosis, the physiology of the coagulation system, and the impact of this on the pharmacology of antithrombotic agents [17]. And interventions include pharmacologic anticoagulation that places patients at higher risk for bleeding complications, requires frequent laboratory safety monitoring, and typically involves injections [18], which increases not only clinical workload but also patients’ suffering.

Currently, due to the lack of evidence supporting the safety and effectiveness of mechanical or pharmacological interventions in preventing venous thromboembolism in children, addressing risk factors has become a primary strategy to reduce its incidence [19]. It has been estimated that best practice prophylaxis may reduce the incidence of DVT by up to 70% [20]. Proper risk prediction can quickly and accurately identify high-risk children and provide a reference for the implementation of targeted prevention [21,22].

In recent years, many studies have been conducted on the development of risk prediction models for VTE in children. For example, Kerris EWJ et al. [23] developed a predictive algorithm with four predictors and achieved a prediction accuracy of 83%. Walker SC et al. [24] conducted a multi-center retrospective case-control study and proposed a model with the negative predictive value of 99.5%. Jeffray J et al. [25] constructed a model through a study with a sample size of 1567,incorporating 11 predictors, achieved an accuracy rate exceeding 80.0%.

Despite numerous attempts to develop predictive models for VTE in children, the conclusions remain inconsistent and research in this area is relatively limited in China. The objective of this protocol is to describe the methodology for a systematic review and critical appraisal of existing prediction models for pediatric VTE, with the aim of summarizing current evidence and identifying knowledge gaps to inform future research and potential application in specific settings, including China, and to offer a theoretical foundation for improving the prevention of pediatric VTE [26].

## Methods and analysis

This systematic review protocol was registered with the International Prospective Register of Systematic Reviews (PROSPERO) on November 17, 2025 (registration number: CRD420251231316; available at: https://www.crd.york.ac.uk/PROSPERO/

view/CRD420251231316). The protocol adheres to the Preferred Reporting Items for Systematic Reviews and Meta-Analyses Protocols (PRISMA-P) guidelines [27] (see S1 Appendix). A rigorous methodological framework is employed, utilizing the Critical Appraisal and Data Extraction for Systematic Reviews of Prediction Modeling Studies (CHARMS) checklist to guide the evaluation and data extraction of prediction modeling studies [28] (S2 Appendix). Risk of bias in the included prediction models will be assessed using the Prediction Model Risk of Bias Assessment Tool (PROBAST) [29] (S3 Appendix). Additionally, reporting of this protocol follows the Transparent Reporting of a multivariable prediction model for Individual Prognosis Or Diagnosis (TRIPOD) statement for multivariable prediction models [30] (S4 Appendix).

### Eligibility criteria

With the guidance of PICOTS (population, index, comparator, outcome, timing, setting) framework [31] we set the eligibility criteria of our study, as listed in Table 1. The selection process of this study will be conducted according to this eligibility criteria.

**Table 1:** Eligibility criteria for the systematic review framed according to the PICOTS system.

Item	Definition
**Population**	*Pediatric patients*
**Index**	*Any diagnostic prediction model to predict the risk of VTE in children*
**Comparator**	*Not applicable*
**Outcome**	*VTE*
**Timing**	*VTE occurred among children at any time*
**Setting**	*Settings including wards, pediatric intensive care units, neonatal intensive care units, outpatients and other healthcare institutions*

### Population

Studies developing or validating prediction models for venous thromboembolism (VTE) in patients under 18 years of age will be taken into consideration for inclusion.

### Intervention

Studies on diagnostic prediction models designed to help physicians diagnose pediatric VTE will be considered for inclusion in this systematic review.

### Outcome

The focus of this study is pediatric VTE, which is unpredictable among children, and there is no standard in prevention. Since VTE can occur at any age during childhood, all the studies related to VTE in children will be included, and the subject age of each study will be listed in tables. Discriminative and calibration performance of each risk prediction models will be assessed.

### Timing

Studies focusing on pediatric VTE among children at any age under 18 years old will be included.

### Setting

This review includes prediction models that are intended to be used clinically in multiple settings, such as inpatient and outpatient hospital departments and healthcare institutions across different levels of care.

### Types of studies and limits

All study designs are eligible for this review, which encompasses every type of research on prediction models for pediatric VTE, aside from animal studies. Published literature through April 20, 2026, will be considered.

### Search methods for identification of studies

#### Information sources.

The search strategy includes querying PubMed, Web of Science, Cochrane Library, CNKI, and Wanfang. Manual reference list reviews of the included literature will also be undertaken to identify additional potentially pertinent studies.

#### Search strategy.

To ensure thorough literature coverage, this study will follow a three‑step search approach. Step one involves two experienced researchers developing the search strategy, including search terms for thromboembolism, predictive modeling, and pediatrics. An initial search will be performed in PubMed and CNKI, and the terms will be further refined in consultation with a pediatric specialist. In step two, two independent researchers will carry out the complete searches. Step three consists of a detailed review of the bibliographies of included articles to locate further studies that meet the inclusion criteria.

### Data collection and analysis

#### Data management.

Endnote X9 will be used to import all bibliographic records. Its built-in duplicate detection features will be applied to identify and remove duplicate records.

#### Selection process.

Two researchers will carry out the screening process independently. In the initial stage, both will independently review the titles and abstracts to identify potentially relevant studies. Studies that are uncertain or possibly relevant, including conference papers lacking abstracts, will be kept for subsequent full-text review. In the following stage, both researchers will independently read the full texts of the retrieved studies and determine eligibility based on predefined criteria. In case of disagreement, a third researcher will be consulted as needed. The selection process will be documented according to the PRISMA guidelines (See Fig 1).

**Fig 1:**
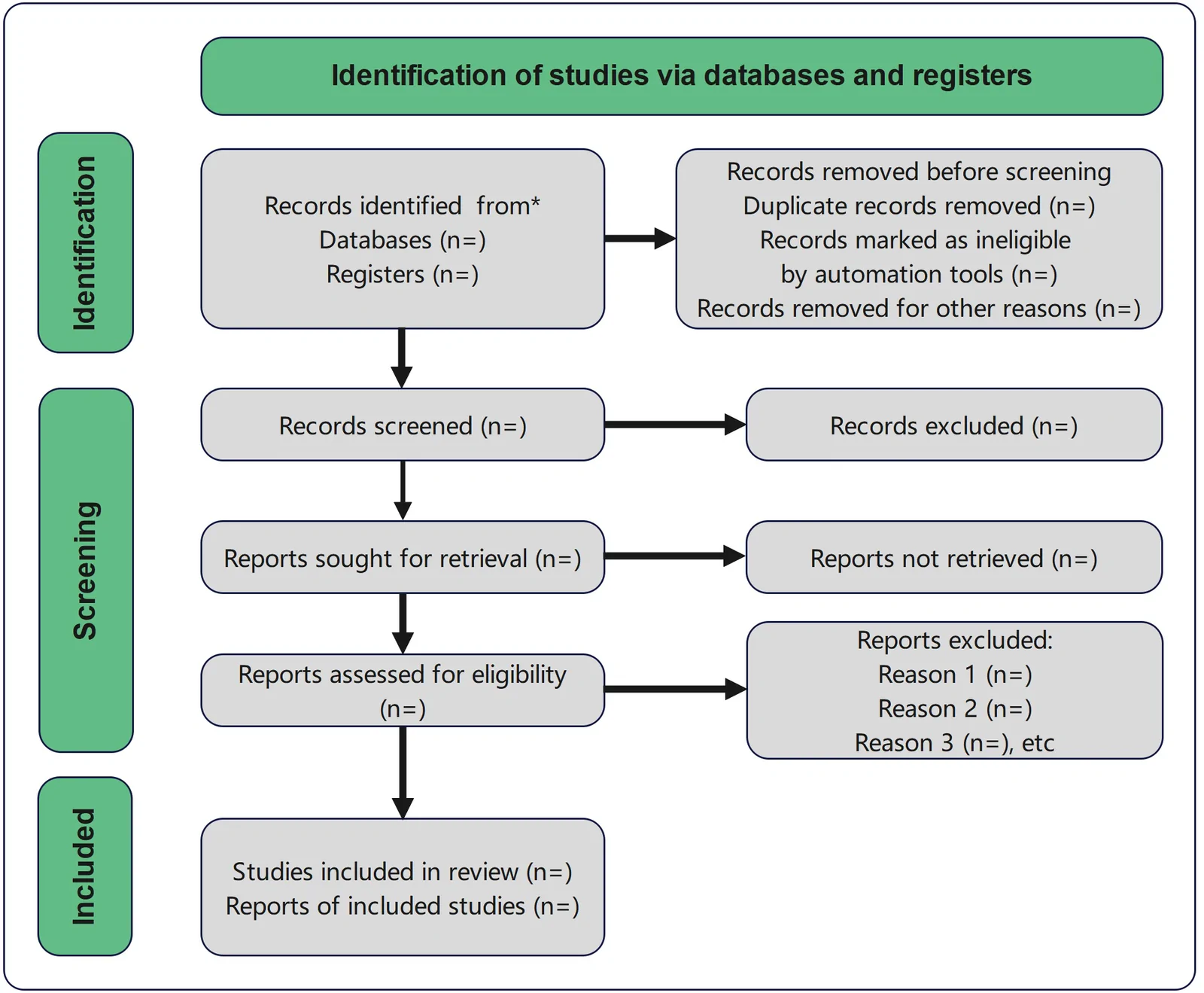
Flow chart of selection process.

#### Data extraction.

Two independent reviewers will conduct data extraction from selected studies using a standardized electronic Excel spreadsheet. Based on the CHARMS checklist, the Excel sheets cover 11 domains such as target information, data sources, participants, predicted outcomes, candidate predictors, sample size, and missing data [28]. The evaluation of model performance metrics should encompass not only discrimination and calibration, but also the outcomes derived from decision curve analysis and net benefit assessments [32,33]. Finally, the researchers will cross-check the extracted data and review the full text to identify and correct discrepancies, ensuring accuracy.

#### Critical appraisal.

The PROBAST tool will be employed for a critical assessment of bias risk and applicability within the selected risk prediction modeling studies [29]. This assessment will encompass four primary domains: the study population, predictor variables, outcomes, and the statistical analyses, with a total of 20 items. Two independent researchers will conduct the assessments, and any discrepancies will be resolved by a third researcher’s decisive judgment. Furthermore, a detailed tabulation will display the findings from the bias risk and applicability assessments.

#### Qualitative and quantitative data synthesis of prediction models.

Data from included studies on prediction models will be systematically tabulated to facilitate the comparison of predicted survival outcomes, final model predictors, and associated performance metrics [28]. When available, uncertainty measures for these metrics will be reported or estimated through established methods [31].

#### Reporting and presentation of findings.

Systematic review will be reported in accordance with the Preferred Reporting Items for Systematic Reviews and Meta-Analyses (PRISMA) guidelines [34] and the TRIPOD statement [30] to improve report transparency and completeness.

#### Discussion and conclusion.

The identification of risk factors and the development of effective prevention strategies for pediatric VTE have been pursued across numerous studies over the past two decades. Yet inconsistent findings have precluded the establishment of a unified approach to risk stratification in children. Unlike in adult populations, where validated tools such as the Caprini and Padua scores are widely adopted, pediatric VTE risk prediction faces domain-specific challenges that warrant dedicated investigation.

First, the low incidence of VTE in children, combined with heterogeneity in underlying conditions (e.g., congenital heart disease, malignancy, trauma), has resulted in small-sample, single-center studies with limited generalizability. Reported performance metrics—such as area under the curve, sensitivity, and specificity—often reflect over-optimistic estimates that lack external validation. Second, even when similar predictors are examined, operational definitions and measurement methods vary considerably across studies (e.g., definitions of “major surgery” or “central venous catheter-associated thrombosis”), complicating direct comparisons and evidence synthesis. Third, the wide pediatric age range—from neonates to adolescents—introduces developmental variations in hemostasis that are not captured by adult-derived tools, further challenging model transportability.

While methodological frameworks for systematic reviews and prediction model appraisal are well established (e.g., PRISMA, TRIPOD, PROBAST), their application to pediatric VTE literature poses unique challenges not fully addressed by generic guidelines. To our knowledge, no previous systematic review has comprehensively appraised pediatric VTE prediction models with explicit attention to these domain-specific methodological issues.

The present protocol offers a structured approach to systematically identifying, appraising, and synthesizing existing evidence. We will critically examine methodological quality using PROBAST, document the extent of external validation, identify consistently associated predictors, and explore sources of heterogeneity through subgroup analyses. For clinicians, this review will provide an evidence base to support informed decision-making. For researchers, it will identify current evidence gaps—particularly the scarcity of externally validated models and the need for standardized predictor definitions—offering clear direction for future model development and validation efforts.

## Strengths and limitations

This study will be conducted in accordance with the guidelines of the Cochrane Handbook and aims to provide high-quality evidence on prediction models for pediatric VTE. By describing in detail the basic characteristics, predictors, and predictive performance of different prediction models, this study will help clinical physicians to select appropriate prediction models to prevent the occurrence of VTE in pediatric patients. This systematic review of prediction models will not only facilitate the selection of the optimal model but also clarify its applicability and generalizability, establishing a basis for further evaluation and validation [31,35].

However, this systematic review will primarily conduct qualitative descriptive analyses without merging model parameters. Variations in clinical backgrounds, patient baseline characteristics, and statistical methodologies across the studies are likely to contribute to increased heterogeneity. Overall, this study will offer a thorough assessment of models published for VTE prediction.

## Supporting information

S1 Appendix Preferred Reporting Items for Systematic Review and Meta -Analysis Protocols (PRISMA-P). (DOCX)

S2 Appendix Critical appraisal and data extraction for systematic reviews of prediction modeling studies (CHARMS). (PDF)

S3 Appendix Prediction Model Risk of Bias Assessment Tool (PROBAST). (PDF)

S4 Appendix Transparent Reporting of a multi-variable prediction model for Individual Prognosis Or Diagnosis (TRIPOD). (PDF)
