# Supplementary material for: Prediction models for venous thromboembolism in pediatric patients: A protocol for systematic review and critical appraisal
Source: PLoS One. 2026 Nov 4;21(11):e0361300. doi: 10.1371/journal.pone.0361300 (PMC13734588; doi:10.1371/journal.pone.0361300)
Supplement: Supplementary file 1 [file pone.0361300.s002.pdf]

OPEN ACCESS Freely available online

PLOS | MEDICINE

Guidelines and Guidance

# Critical Appraisal and Data Extraction for Systematic Reviews of Prediction Modelling Studies: The CHARMS Checklist

**Karel G. M. Moons[1¶]*, Joris A. H. de Groot[1¶], Walter Bouwmeester[1], Yvonne Vergouwe[1], Susan Mallett[2], Douglas G. Altman[3], Johannes B. Reitsma[1], Gary S. Collins[3]**

**1** Julius Center for Health Sciences and Primary Care, UMC Utrecht, Utrecht, The Netherlands, **2** Department of Primary Care Health Sciences, New Radcliffe House, University of Oxford, Oxford, United Kingdom, **3** Centre for Statistics in Medicine, University of Oxford, Botnar Research Centre, Windmill Road, Oxford, United Kingdom

## Introduction

Prediction models, both diagnostic and prognostic, are becoming increasingly abundant in the medical literature [1–3]. Diagnostic models are aimed at calculating the probability that an individual has a certain disorder, such as deep vein thrombosis [4,5], ankle fractures [6], or conjunctivitis [7]. Prognostic prediction models concern the prediction of the probability or risk of the future occurrence of a particular outcome or event in individuals at risk of such an event. Prognostic models may involve models for individuals with a particular health condition, such as prediction of recurrence or death after diagnosis of breast cancer [8] or mortality after cardiac surgery [9], but also includes models for predicting the occurrence of future outcomes in apparently healthy individuals such as the risk of developing a coronary event [10] or type 2 diabetes mellitus [11].

There are over 100 models for predicting outcome after brain trauma [12], over 60 models for breast cancer prognosis [13], 45 models for cardiovascular events after being diagnosed with diabetes [14], 43 models for predicting prevalent and incident type 2 diabetes [15], and 20 models for predicting prolonged intensive care stay after cardiac surgery [16]. Furthermore, prediction models are increasingly being appraised and recommended for formal risk assessment in clinical guidelines [17,18].

To evaluate the proliferation of prediction models, systematic reviews are necessary and led to the formation of the Cochrane Collaboration Prognosis Reviews Methods Group [19,20]. Since then, search strategies for identifying prognostic and diagnostic prediction model studies have been developed [21–23], validated, and further refined [24].

However, no published checklists support the design of systematic reviews of prediction modeling studies, or what to extract and how to appraise primary prediction modelling studies. Existing guidance for synthesizing studies of prognostic factors [25,26] does not address studies of multivariable prediction models. Instead, reviews of prediction model studies have created their own checklist [2,12,14,15,27–30], with variable inclusion of key details.

Our aim was to design a **CH**ecklist for critical **A**ppraisal and data extraction for systematic **R**eviews of prediction **M**odelling **S**tudies (CHARMS). The checklist is designed to help form a review question for and appraisal of all types of primary prediction modelling studies, including, regressions, neural network, genetic programming, and vector machine learning models [1–3,12,14,15,27–30]. Some items, such as "selection of predictors during multivariable modelling" and "model presentation", are somewhat more specific to regression approaches. The checklist is not intended for systematic reviews of primary studies of prognostic factors, for which we refer to the QUIPS tool [25,26], nor is it intended for prediction model impact studies in which, in principle, a comparative (intervention) design is used

The **Guidelines and Guidance** section contains advice on conducting and reporting medical research.

**Summary Points**

- Publications on clinical prediction models have become abundant for both prognostic and diagnostic purposes. Systematic reviews of these studies are increasingly required to identify and critically appraise existing evidence.
- No specific guidance exists to help frame a well-defined review question and determine which details to extract and critically appraise from primary prediction modelling studies.
- Existing reporting guidelines, quality assessment tools, and key methodological publications were examined to identify seven items important for framing the review question and 11 domains to extract and critically appraise the primary included studies.
- Together these items and domains form the **CH**ecklist for critical **A**ppraisal and data extraction for systematic **R**eviews of prediction **M**odelling **S**tudies (CHARMS).

**Citation:** Moons KGM, de Groot JAH, Bouwmeester W, Vergouwe Y, Mallett S, et al. (2014) Critical Appraisal and Data Extraction for Systematic Reviews of Prediction Modelling Studies: The CHARMS Checklist. PLoS Med 11(10): e1001744. doi:10.1371/journal.pmed.1001744

**Published** October 14, 2014

**Copyright:** © 2014 Moons et al. This is an open-access article distributed under the terms of the Creative Commons Attribution License, which permits unrestricted use, distribution, and reproduction in any medium, provided the original author and source are credited.

**Funding:** We gratefully acknowledge financial contribution by the Netherlands Organisation for Scientific Research (project 918.10.615). The funders had no role in study design, data collection and analysis, decision to publish, or preparation of the manuscript.

**Competing Interests:** The authors have declared that no competing interests exist.

* Email: k.g.m.moons@umcutrecht.nl

¶ Moons and de Groot contributed equally to this work and are joint first authors.

**Provenance:** Not commissioned; externally peer reviewed

**Box 1. Types of Prediction Modelling Studies**

- *Prediction model development studies without external validation* aim to develop a prognostic or diagnostic prediction model from the dataset at hand: the development set. Such studies commonly aim to identify important predictors for the outcome under study, assign mutually adjusted weights per predictor in a multivariable analysis, develop a final prediction model, and quantify the predictive performance (e.g., discrimination, calibration, classification) of that model in the development set. As model overfitting may occur, particularly in small datasets, development studies ideally include internal validation using some form of data re-sampling techniques, such as bootstrapping, jack-knife, or cross-validation, to quantify any optimism in the predictive performance of the developed model.
- *Prediction model development studies with external validation in independent data* have the same aim as the previous type, but the development of the model is followed by quantifying the model's predictive performance in participant data external to the development dataset. This may be done in participant data collected by the same investigators, commonly using the same predictor and outcome definitions and measurements, but from a later time period (temporal or narrow validation), or by other investigators in another hospital or country (geographical or broad validation).
- *External model validation studies with or without model updating* aim to assess and compare the predictive performance of an existing prediction model using new participant data that were not used to develop the prediction model and possibly adjust or update the model in case of poor performance based on the validation data.

Prediction studies exploring which predictors independently contribute to the prediction of a particular prognostic or diagnostic outcome as well as studies aimed at quantifying the impact of using a prediction model (on, e.g., clinical decision making, patient outcomes, or cost-effectiveness of care) relative to not using the model may also be considered in a systematic review of prognostic and diagnostic prediction models [2]. However, data extraction and critical appraisal of those types of prediction studies is very different as they have different aims, designs, and reporting issues compared to studies developing or validating prediction models. Therefore, here we explicitly focus on reviews of studies aimed at developing, validating, or updating a prediction model.

[1,31,32]. Box 1 shows the types of prediction modelling studies for which the CHARMS checklist was developed.

## Development of the Checklist

We developed our checklist based on published risk of bias tools, existing critical appraisal checklists for systematic reviews of randomised therapeutic trials and diagnostic test accuracy research, methodological recommendations for conduct and reporting of prediction model research, and data extraction sheets used in published reviews of prediction modelling studies after contacting authors.

First, we reviewed the existing reporting guidelines for other types of clinical research including CONSORT, REMARK, STARD, STROBE, GRIPS [33–37] and for the reporting of systematic reviews (PRISMA) [38]. Furthermore, we considered existing quality assessment tools including the Cochrane Risk of Bias tool [39] for randomised therapeutic studies, QUADAS (and QUADAS-2) for diagnostic accuracy studies [40,41], and the QUIPS checklist for appraisal of prognostic factor studies [25,26]. We then reviewed published systematic reviews of prediction models and prognostic factor studies, along with the checklists or quality appraisal criteria used in those reviews [12,27–29,42–46]. Finally, we identified key methodological literature discussing recommended approaches for the design, conduct, analysis, and reporting of prediction models, followed by a search of the corresponding reference lists [3,19,31,32,37,47–59].

Initial pilot versions of this checklist were presented and discussed at the annual Cochrane Prognosis Methods Group meetings and workshops from 2010–2014, held during the Cochrane Collaboration Colloquia, and modified based on feedback received during these meetings. Consecutive iterations of the checklist were applied, tested, and modified in various systematic reviews of prediction models [2,14–16,29,60–62], which ultimately led to the current checklist. For the actual reporting of systematic reviews of prediction models, we refer to the PRISMA statement [38].

## The Checklist

The checklist contains two parts. Table 1 summarises key items to guide the framing of the review aim, search strategy, and study inclusion and exclusion criteria. Table 2 and Text S1 describe the overall domains and specific items within each domain to extract from the reports of primary prediction modelling studies in light of the review question, with a view to evaluate risk of bias and applicability.

*Risk of bias* refers to the extent that flaws in the design, conduct, and analysis of the primary prediction modelling study lead to biased, often overly optimistic, estimates of predictive performance measures such as model calibration, discrimination, or (re)classification (usually due to overfitted models). *Applicability* refers to the extent to which the primary study matches the review question, and thus is applicable for the intended use of the reviewed prediction model(s) in the target population.

### Guidance to frame the review question, search strategy, and study inclusion and exclusion criteria

Table 1 addresses seven key issues (i.e., prognostic versus diagnostic prediction model, intended scope of the review, type of prediction modelling studies [see also Box 1], target population to whom the prediction model applies, outcome to be predicted, time span of the prediction, and intended moment of using the model) that are helpful for systematic reviewers to frame the review question and design the review. A focused review question enables researchers to develop a tailored search strategy and to define the inclusion and exclusion criteria—and thus the applicability—of primary studies included in the review.

At the outset, the reviewer should decide whether the aim is to review prognostic or diagnostic models (item 1) and define the scope of the review (item 2). It is then important to decide whether to include model development studies, model validation studies, or both (item 3 and Box 1). For example, if the review aims to assess the performance of a specific prediction model, then only external validation studies of that model are applicable for the review.

Defining the target population of the prediction model(s) under review (item 4) and the outcome(s) to be predicted (item 5) are related items that are particularly important to indicate the

**Table 1.** Key items to guide the framing of the review aim, search strategy, and study inclusion and exclusion criteria.

| Item | Comments and examples |
|---|---|
| **1. Prognostic versus diagnostic prediction model** | Define whether the aim is to review models to predict: |
| | • Future events: prognostic prediction models |
| | • Current (disease) status: diagnostic prediction models |
| **2. Intended scope of the review** | Define intended scope of the review and intended purpose of the models reviewed in it. Examples: |
| | • Models to inform physicians' therapeutic decision making |
| | • Models to inform referral to or withholding from invasive diagnostic testing |
| **3. Type of prediction modelling studies (see also Box 1)** | Define the type of prediction modelling studies to include. Examples of study types (Box 1): |
| | • Prediction model development without external validation in independent data |
| | • Prediction model development with external validation in independent data |
| | • External model validation, possibly with model updating |
| **4. Target population to whom the prediction model applies** | Define the target population relevant to the review scope. Examples: |
| | • Women with diagnosed breast cancer |
| | • Healthy adult men in the general population |
| **5. Outcome to be predicted** | Define the outcome of interest to be predicted: |
| | • Specific future event, such as a fatal or non-fatal coronary heart disease |
| | • Specific diagnostic target disease, such as presence of lung embolism |
| **6. Time span of prediction** | Define over what specific time period the outcome is predicted (prognostic models only). Example: |
| | • Event within a specific time interval, such as event within 3 months, 1 year, or 10 years |
| **7. Intended moment of using the model** | The systematic review may focus on models to be used at a specific moment in time. Examples: |
| | • Models to be used at the moment of diagnosis of a particular disease |
| | • Models to be used preoperatively to predict the risk of postoperative complications |
| | • Models to be used in asymptomatic adults to detect undiagnosed type 2 diabetes mellitus |

doi:10.1371/journal.pmed.1001744.t001

potential usefulness and application of the review results. For example, relevance to physicians and patients is enhanced by models that predict patient-relevant outcomes, such as death, pain, or recurrence of disease, rather than those that predict process outcomes such as duration of hospital stay or intermediate outcomes, except when there is a clear and established causal association with a subsequent patient-relevant outcome (e.g., predicting CD4 count instead of complications in patients with HIV [47]).

Prognostic models commonly have a better predictive accuracy for short-term outcomes than for long-term outcomes (item 6) [63]. However, predicting long-term outcomes may sometimes be more relevant from a patient perspective, though this is obviously questionable in very elderly individuals [64].

Finally, clarifying when the model is intended to be used is important to define what sorts of models are relevant for the review (item 7). Models that incorporate predictors collected after this predefined time point are inappropriate. For example, if the aim is to review prognostic models to preoperatively predict the risk of developing post-operative pain within 48 hours after hip surgery, studies including intraoperative characteristics are not useful.

In Box 2 we give various examples of potential review questions of both prognostic and diagnostic models.

## Relevant items to extract from individual studies

The key items to be extracted from each primary study are grouped within 11 domains. Similar to critical appraisal checklists for systematic reviews of randomised therapeutic and diagnostic accuracy studies, these address potential sources of bias in the primary studies and issues that may affect the applicability of the results in relation to the intended use of the prediction models.

**Source of data.** Data from cohort, nested case-control, or case-cohort studies are recommended for prognostic model development and validation studies, and cross-sectional designs for diagnostic modelling studies [47,58,59,65–67]. Clearly, a prospective cohort design is preferable, as it enables optimal measurement of predictors and outcome. However, prospective studies evaluating (validating) the performance of an existing model predicting a long-term outcome, e.g., ten-year survival, may be too costly or the results insufficiently timely. Retrospective cohorts typically have a longer follow-up period, but usually at the expense of poorer data quality and unmeasured predictors [13]. A non-nested case-control design, as opposed to a nested case-control or case-cohort design, is inappropriate for developing a prediction model since the design does not enable calculation of absolute risks and thus yields incorrect estimates of model intercept or baseline hazard [65–68].

Randomised trials are a specific form of a prospective cohort study and thus share its advantages. However, restrictive eligibility criteria for entry into the trial may hamper generalizability of the prediction model. Furthermore, treatments shown to be effective in the trial should be acknowledged and possibly accounted for in the prediction model, as they may affect the predictive accuracy of the prognostic model [47,56]. Finally, data from existing registries (e.g., administrative or routine care hospital databases) are increasingly used in prediction modelling studies. However, such databases are especially prone to missing

**Table 2.** Relevant items to extract from individual studies in a systematic review of prediction models for purposes of description or assessment of risk of bias or applicability.

| Domain | Key items | General | Applicability | Risk of bias |
|---|---|---|---|---|
| **Source of data** | • Source of data (e.g., cohort, case-control, randomised trial participants, or registry data) | | X | X |
| **Participants** | • Participant eligibility and recruitment method (e.g., consecutive participants, location, number of centres, setting, inclusion and exclusion criteria) | X | X | |
| | • Participant description | X | X | |
| | • Details of treatments received, if relevant | | X | X |
| | • Study dates | X | X | |
| **Outcome(s) to be predicted** | • Definition and method for measurement of outcome | | X | X |
| | • Was the same outcome definition (and method for measurement) used in all patients? | | | X |
| | • Type of outcome (e.g., single or combined endpoints) | X | X | |
| | • Was the outcome assessed without knowledge of the candidate predictors (i.e., blinded)? | | | X |
| | • Were candidate predictors part of the outcome (e.g., in panel or consensus diagnosis)? | | | X |
| | • Time of outcome occurrence or summary of duration of follow-up | | X | |
| **Candidate predictors (or index tests)** | • Number and type of predictors (e.g., demographics, patient history, physical examination, additional testing, disease characteristics) | X | | |
| | • Definition and method for measurement of candidate predictors | | X | X |
| | • Timing of predictor measurement (e.g., at patient presentation, at diagnosis, at treatment initiation) | | X | |
| | • Were predictors assessed blinded for outcome, and for each other (if relevant)? | | | X |
| | • Handling of predictors in the modelling (e.g., continuous, linear, non-linear transformations or categorised) | | | X |
| **Sample size** | • Number of participants and number of outcomes/events | X | | |
| | • Number of outcomes/events in relation to the number of candidate predictors (Events Per Variable) | | | X |
| **Missing data** | • Number of participants with any missing value (include predictors and outcomes) | X | | X |
| | • Number of participants with missing data for each predictor | | | X |
| | • Handling of missing data (e.g., complete-case analysis, imputation, or other methods) | | | X |
| **Model development** | • Modelling method (e.g., logistic, survival, neural networks, or machine learning techniques) | X | | |
| | • Modelling assumptions satisfied | | | X |
| | • Method for selection of predictors for inclusion in multivariable modelling (e.g., all candidate predictors, pre-selection based on unadjusted association with the outcome) | | | X |
| | • Method for selection of predictors during multivariable modelling (e.g., full model approach, backward or forward selection) and criteria used (e.g., p-value, Akaike Information Criterion) | | | X |
| | • Shrinkage of predictor weights or regression coefficients (e.g., no shrinkage, uniform shrinkage, penalized estimation) | | X | X |
| **Model performance** | • Calibration (calibration plot, calibration slope, Hosmer-Lemeshow test) and Discrimination (C-statistic, D-statistic, log-rank) measures with confidence intervals | | X | |
| | • Classification measures (e.g., sensitivity, specificity, predictive values, net reclassification improvement) and whether a priori cut points were used | | | X |
| **Model evaluation** | • Method used for testing model performance: development dataset only (random split of data, resampling methods, e.g., bootstrap or cross-validation, none) or separate external validation (e.g., temporal, geographical, different setting, different investigators) | | | X |

**Table 2.** Cont.

| Domain | Key items | General | Applicability | Risk of bias |
|---|---|---|---|---|
| | • In case of poor validation, whether model was adjusted or updated (e.g., intercept recalibrated, predictor effects adjusted, or new predictors added) | | X | X |
| **Results** | • Final and other multivariable models (e.g., basic, extended, simplified) presented, including predictor weights or regression coefficients, intercept, baseline survival, model performance measures (with standard errors or confidence intervals) | X | X | |
| | • Any alternative presentation of the final prediction models, e.g., sum score, nomogram, score chart, predictions for specific risk subgroups with performance | X | X | |
| | • Comparison of the distribution of predictors (including missing data) for development and validation datasets | | | X |
| **Interpretation and Discussion** | • Interpretation of presented models (confirmatory, i.e., model useful for practice versus exploratory, i.e., more research needed) | X | X | |
| | • Comparison with other studies, discussion of generalizability, strengths and limitations | X | X | |

doi:10.1371/journal.pmed.1001744.t002

data and missing important predictors, which can affect the predictive accuracy and applicability of the resulting prediction model [31,56,58,69].

**Participants.** The participant recruitment method is important to establish whether the study population is representative of the target population. A review of 83 diagnostic prediction models for detection of ovarian malignancy found that studies often sampled participants non-consecutively [70], increasing the risk of bias due to selective sampling [42,56,71]. Also, it is important to ascertain from the publication whether all included participants were eventually used to develop or validate the prediction model [15,56]. Selective inclusion based on data availability is likely to influence the predictive accuracy of the prediction model, as study data are seldom missing completely at random but are often missing in a selective and biased way (see section below on missing data).

Participant description, including inclusion and exclusion criteria, study setting (e.g., primary or secondary care), and number of centres, is important to allow for proper assessment of the applicability and thus generalizability of the study findings [40,41,56,72]. For reviews of a single model that has been validated in different study samples, differences or heterogeneity in study design, sample characteristics, and setting that will affect the performance of the prediction model should be determined. For example, prediction models developed in secondary care perform less well when evaluated in a primary care setting [73,74]. Reviews of prognostic models for patients with breast cancer [56] and patients with lower back pain [75] have identified that participant characteristics were often poorly reported.

The performance of prediction models may also vary depending on whether the study participants have received any treatment (including self-administered interventions) that may modify the outcome occurrence. It is therefore important to determine whether the review addresses treated or non-treated individuals or both, and whether the treatment effects (i.e., treatment predictors) were handled appropriately in the models. Finally, the dates of participant recruitment provide important information on the technological state of the tests and treatments used, and the lifestyle factors at that time. The predictive accuracy of models may change over time and require periodic updating [76], as was done for the QRISK models [77].

**Outcome to be predicted.** The definition and measurement of the outcome event (prognostic models) or the target disease (diagnostic models) in the primary studies should correspond to the outcome definition of the systematic review question. Different outcome definitions and measurement methods may lead to differences in study results and are a source of heterogeneity across studies and thus risk of bias. Occasionally a different definition of outcome is intentional to examine the usefulness of a model to predict alternative outcomes. For example, one may intentionally seek to validate for non-fatal events a model originally developed for predicting fatal events. A review of cancer prognostic models found that outcomes were poorly defined in 40% of the studies [60,78]. It was often unclear whether mortality referred to cancer mortality or overall mortality from any cause, and in the definition of disease-free survival it was unclear which events were included.

In diagnostic modelling studies, establishing the presence or absence of the target disease is known as verification by a reference standard. Primary studies on the same target disease frequently use different reference standards which may have different accuracy for determining the true target disease status, potentially compromising the validity of study results; using a suboptimal reference standard may lead to misclassification of the target disease [79–81].

Some modelling studies use a combined outcome; for example, cardiovascular disease often comprises myocardial infarction, angina, coronary heart disease, stroke, and transient ischaemic stroke. A combined outcome is considered easily translatable to clinical practice or to achieve a higher effective sample size, but could lead to important predictors not being identified, as predictors may have opposite predictive effects in the component outcomes, causing their predictive contributions to cancel each other out. Reviewing and summarising predictors in models using combined outcomes is particularly challenging [82,83]. In studies validating a prediction model for a combined outcome, the number and severity of individual component outcomes may differ markedly from the derivation study, potentially affecting the predictive accuracy of the model in the validation dataset [84]. When available, the systematic review should record the frequency of the individual components in the combined outcome to enable comparison across studies. If this information is not reported in the

## Box 2. Examples of Systematic Reviews of Prognostic or Diagnostic Prediction Models with Different Aims

**Reviews of prediction models for specific target populations (development and/or validation)**

- Existing models for predicting the risk of having undiagnosed or developing (incident) type 2 diabetes in adults [15].
- Prognostic models for activities of daily living, to be used in the early post-stroke phase [46].

**Reviews of prediction models for specific outcomes in a target population (development and/or validation)**

- Prognostic models for survival, for independence in activities of daily living, and for getting home, in patients with acute stroke [27].
- Prediction models for diagnosis of venous thromboembolism in patients suspected of having the disease [28].

**Review of the performance of one or more specific models (validation)**

- Predictive performance of the EuroSCORE for operative mortality following cardiac surgery when validated in other patient samples [139].
- Relative predictive performance of specific prognostic models for occurrence of cardiovascular disease when applied in general populations [44].

**Reviews of all existing models in a particular clinical field (development and/or validation)**

- Existing prognostic models in reproductive medicine [29].
- Existing prognostic models in the traumatic brain setting [12].

**Review of methods and reporting of prediction models (development and/or validation)**

- Quality of reporting of diagnostic and prognostic modelling studies published in high impact general medical journals or in a specific time period [2,48].
- Reporting and methods used to develop prognostic models in cancer [60].

**Review of added value of specific predictor or updating of a specific model (development and/or validation)**

- Added predictive value of C-reactive protein to the Framingham risk scores [134].
- Added predictive value of carotid imaging markers to existing cardiovascular predictors in the general population [140].

primary study and cannot be retrieved by contacting the study authors, then this should be reported in the systematic review.

In diagnostic studies the importance of assessing the reference test without knowledge of (i.e., blinded to) the results of the index tests is well established [35,40,54,69,79,80,85]. The same issue is also important in prognostic studies where the assessor of the outcome occurrence should be blinded to ascertainment of the predictor [47,48]. In the absence of blinding, the predictive ability of the model may be overestimated because the predictors may be used in assessing the outcome. Blinded outcome assessment, in both diagnostic and prognostic studies, is most important when outcomes require subjective interpretation (e.g., results from imaging) that could be biased by knowledge of predictors. For so-called "hard" outcomes, such as overall mortality, blinded outcome assessment is less important. However, cause-specific mortality may include subjective interpretation so that knowledge of the predictors could bias outcome assignment. Several reviews have reported that many studies did not blind the outcome measurement for the candidate predictors [70,81,86,87].

A special case of incorporating the predictor information in the outcome assessment is the use of so-called consensus or expert panel outcome assessments. This is often used in diagnostic studies for target diseases where the reference standard used in practice is known to include a subjective assessment of information [52,54,88,89]. Here, a consensus panel typically uses all available information on the study participants, including the predictors (or index tests) under study, to determine whether the target disease is present. The results of the predictors are directly and deliberately incorporated in the assessment of the target condition, usually leading to optimistic predictive accuracy of the developed models. This specific form of non-blinded outcome assessment bias is commonly referred to as "incorporation bias" [52,88,89].

In the prognostic setting, retrieval of the follow-up period or a summary of the follow-up from the primary studies deserves special attention. Disappointingly, these key details are often poorly reported [72,75]. A recent review found the number of participants with ten years follow-up was frequently not reported, even in studies validating prognostic models predicting a ten-year outcome [15].

**Candidate predictors.** Candidate predictors may range from simple patient demographics and clinical characteristics to advanced test results. We emphasise that *candidate* refers to the predictors chosen to be studied for their predictive performance, and not restricted to those included in the multivariable analysis [59]. The number of candidate predictors analysed in the primary studies is highly important. Together with the number of participants with the outcome (i.e., those with the event or the target disease) they contribute to the assessment of whether the model is likely to be overfitted. Overfitting occurs when idiosyncratic features of the development data attain spurious statistical significance and are retained in the final model: the model is too closely tailored to the data at hand [51,58]. These models do not produce inaccurate predictions in the dataset from which they are developed, but they do when applied to other individuals. Predictions tend to be too extreme; low predicted risks will be too low and high predicted risks too high. Overfitting thus leads to models that are not transportable or generalizable.

Different definitions and measurement methods of candidate predictors are a potential source of heterogeneity and thus risk of bias, and the use of different measurement methods may affect the strength of predictors and influence whether the predictors ultimately are included in the prediction model [42,61]. For example, type 2 diabetes mellitus, a known risk factor and therefore predictor for cardiovascular disease, can be defined by an oral glucose tolerance test, HbA1c measurement, fasting plasma glucose measurement, or even by self-report. These different predictors may have different predictive effects in the

multivariable models. Also, models including predictors measured using routinely accessible equipment are likely more generalizable than predictors measured with less available techniques [61]. As with the outcome, the definition and measurement method of the predictors may sometimes be intentionally different when evaluating an existing model in a separate dataset. The review should highlight differences in definitions or measurement methods of any of the predictors, so readers can place the results in context.

Candidate predictors that can vary over time should be available and measured at the time of intended use of the prediction model, not at a later moment in time or after the outcome has occurred [47,90].

As described for outcome assessment, measurement of predictors with knowledge of outcome information may inflate the predictive accuracy of the predictors and thus of the final prediction model. This concern particularly applies to predictors requiring subjective interpretation. In prospective studies, predictor assessment is inherently blinded, as it is completed prior to outcome occurrence. It may also be important to blind assessment of predictors to each other, particularly if a review seeks to address the predictive contribution of an additional subjective predictor beyond previously obtained predictors. For example, if the predictive ability of an MRI in addition to laboratory measurements is studied, the MRI should be interpreted blinded to the laboratory measurements to reduce possible bias [73,91].

The methods used to handle predictors in the analysis can influence which predictors are selected for inclusion in the model and so affect model predictions. Continuous or categorical predictors are frequently dichotomised for the analysis [2,42,56,60,78] despite strong evidence and recommendations to the contrary [92–94]. Categorisation assumes a constant risk up to the cut-point and then a different risk beyond the cut-point, which is implausible and nonsensical. In addition, dichotomising discards information and commonly results in a loss of power [93]. Dichotomising predictors, particularly when choosing a so-called "optimal" cut point based on data from one study, often causes selection of spurious predictors and overfitting, reducing the reliability and applicability of model predictions in new patients [55,93–96].

**Sample size.** One of the biggest concerns when developing a prediction model is the risk of overfitting. For dichotomous outcomes, overfitting typically arises when the number of individuals with the outcome (event or target disease) of interest is small relative to the number of variables. The number of variables includes all candidate predictors, transformations for continuous predictors, indicator variables for categorical predictors, and interactions examined. The number of events-per-variable (EPV) is commonly used to calculate the sample size, where attaining a sample size with an EPV of ten or more is frequently recommended to avoid overfitting [97–101]. For studies validating prediction models, sample size considerations are not well established, but a minimum of 100 events and 100 non-events have been suggested [102]. For continuous outcomes, 20 participants per predictor have been recommended [51].

A systematic review should therefore record both the number of individuals in the study and the number of individuals with the outcome or target disease. Numerous systematic reviews of prediction models have reported that the number of events per candidate predictor is often poorly reported and, when it is reported, that the EPV is often less than ten [2,15,56,78].

**Missing data.** In all types of medical studies, including prediction modelling, some data is not available or not recorded. Differences between studies in the extent and type of missing data and the methods used to handle this missing data may greatly influence model development and predictive performance. Knowing the number of participants with any missing data across all included studies and whether these participants were included in model development or validation is important to understanding possible biases in prediction modelling studies. However, reporting on the frequency and type of missing data is often poor [2,15,56,62,78,103–105] despite the adverse effects of missing data on development, validation, and updating of a prediction model [34,103,105–112]. These adverse effects are related to the amount of missing data [112] and the extent to which data are missing completely at random [108,111]. Missing data are seldom missing completely at random; the missing data are often related to other observed participant data. Consequently, participants with completely observed data are different from those with missing data. A so-called complete-case analysis, which simply deletes participants with a missing value, thus leaves a non-random subset of the original study sample, yielding invalid predictive performance, both when developing and when validating a prediction model. Only if omitted participants are a completely random subset of the original study sample will the estimated predictor-outcome associations and predictive performance measures of the prediction model be unbiased [113]. Multiple imputation is generally acknowledged as the preferred method for handling missing data in prediction research. In this strategy, missing observations are substituted by plausible estimated values derived from analysis of the available data. However, when data are "missing not at random", i.e., missing data is still partly due to unobserved data or characteristics of the participants, multiple imputation does not sufficiently solve the invalidity problem [107,112,113].

Detailed reporting in primary studies on whether missing data may reasonably be missing at random (by comparison of the participants with and without missing values) is invaluable for reviewers to judge the potential for bias. Numerous recommendations for reporting missing data in medical research have been proposed [103,104,114,115]. It is therefore important during the systematic review to record from the primary studies whether the presence of missing data (how much and how handled) was mentioned.

**Model development.** In appraising studies that include model development, first the type of model (e.g., logistic, survival, machine learning, other models) used should be assessed. It is important to summarise and understand key components that might lead to bias and variability between models. An important source of bias in model development is in the method of selecting the final predictors, especially in studies with a small sample size. We split the selection of predictors into two components, the selection of predictors for inclusion in the multivariable analysis and selection during multivariable modelling. The use of different predictor selection methods and criteria for predictor inclusion across studies may yield different models and different amounts of bias. These issues should thus be carefully documented during the review.

*Selection of predictors for inclusion in multivariable modelling.* In some model development studies, predictors are selected for inclusion in the multivariable modelling based on the association of each candidate predictor with the outcome. Although common, such screening or pre-selection based on univariable significance testing carries a great risk of so-called predictor selection bias [51,56,58,116]. Predictor selection bias occurs when predictors selected for inclusion in multivariable modeling have a large but spurious association with the outcome. Including such predictors increases the likelihood of overfitting and thus over-optimistic predictions of a model's performance for other individuals.

Furthermore, predictors that show no association with the outcome in univariable analysis because of small sample size may become associated with the outcome after adjustment for other predictors. The risk of predictor selection bias is greater in smaller datasets (when the EPV ratio is small), and when there are notably weak predictors.

Bias in predictor selection may also occur when continuous predictors are categorised. As discussed, it is recommended to keep continuous variables continuous and to check whether nonlinear transformations (e.g., using restricted cubic splines or fractional polynomials) are indicated [45,51,58,93,94,96].

The systematic review should record how many candidate predictors were examined, any methods used to select predictors, and any methods used to transform predictors prior to inclusion in the multivariable analysis to assess risk of bias.

*Selection of predictors during multivariable modelling.* Just as the selection of predictors for inclusion in the multivariable modelling can contribute to optimistic and biased models due to overfitting, so can the selection of predictors during multivariable modelling. There is no consensus on the best method, but certain methods have been shown to be less useful and increase the risk of model overfitting, such as forward selection techniques [51,55,58,117]. Two of the most commonly used methods are the "full model approach", and "backward elimination". The full model approach pre-specifies all predictors in the final model and no predictors are omitted, which avoids predictor selection bias [51,58]. Whilst this approach sounds attractive, it requires substantive prior knowledge about the most promising candidate predictors [59], which is not always straightforward. Backward elimination starts with all candidate predictors in the model and runs a sequence of statistical tests to remove them from or keep them in the model based on a pre-specified criterion. Possible criteria for predictor inclusion include Akaike or Bayesian Information Criterion, the use of a nominal p-value (e.g., $<0.05$ based on the log likelihood ratio test in regression approaches), or using a change in the model's c-index (see below) [58]. The choice of a relatively small nominal significance level for predictor selection (e.g., p-value$<$ 0.05 or even $<0.01$) generates models with fewer predictors, but increases the chance of missing potentially important predictors, while larger levels (e.g., $p<0.20$ or $p<0.25$) increase the risk of selecting less important predictors. In both cases, overfitting may arise, particularly in small datasets [51,55,58,59].

To address possible overfitting of a model, shrinkage techniques can be used to adjust the estimated weights of the predictors. The corresponding adjusted estimates of predictive performance are likely to be closer to the predictive accuracy that will be found when the developed model is applied to other individuals. Hence, studies that develop prediction models that are adjusted or shrunk are less prone to bias. The need for use of shrinkage methods increases with smaller datasets, although in datasets with a low number of EPV, even shrinkage methods cannot account for all bias [51,58,117,118].

Given the strengths and weaknesses of various modelling and predictor selection strategies, the systematic review should record all information on the multivariable modelling, so readers can gain insight into how each model was developed.

**Model performance.** Regardless of the statistical method used to develop or validate the model, various model performance measures such as calibration, discrimination, (re)classification, and overall measures of performance may be used [51,58,117]. Calibration and discrimination should always be recorded when reviewing clinical prediction models. Calibration refers to how well the predicted risks compare to the observed outcomes; preferably this is evaluated graphically by plotting observed against predicted event rates [51,58,119]. For time-to-event models using, e.g., Cox regression, calibration is usually evaluated at specific time points by comparing observed and predicted risks for groups of individuals [119]. Calibration plots are often supplemented by a formal statistical test, the Hosmer-Lemeshow test for logistic regression and its equivalent for Cox regression. However, such tests have frequently been criticised because of the limited statistical power to assess poor calibration and being oversensitive in large samples [58,117,120,121]. Furthermore, the Hosmer-Lemeshow test gives no indication of the direction or magnitude of any miscalibration. Discrimination refers to how well the model differentiates between those with and without the outcome and is typically assessed using the *c*-statistic, which is the equivalent to the area-under-the-curve of a receiver operating characteristic curve. The *c*-statistic should not be used as the only performance measure, however, since it is influenced by the distribution of predictor values and is often insensitive to inclusion of an additional predictor in the model [59,122–125].

Classification measures, notably sensitivity and specificity, may also be presented. However, the use of these measures requires a predefined probability threshold. The same model would show very different sensitivity and specificity depending on the chosen threshold. The reporting of performance based on thresholds chosen from the data itself can produce over-optimistic and biased performance [95].

Reclassification measures, such as net reclassification improvement or index (NRI), evaluate whether a single biomarker has any incremental value to a prediction model [124,126]. Their use has been criticised as they rely on a priori–defined probability thresholds and do not account for difference in consequences of falsely reclassified individuals [122,127,128]. Furthermore, NRI is a measure of comparative performance and is therefore not directly useful as a measure of performance of a single model.

Recent systematic reviews have found the reporting of performance measures to be poor, with reliance on measures of discrimination [2,15,60]. Objective evaluation across multiple studies and models is difficult if other aspects of model performance are missing. Systematic reviews should ensure that if possible, at a minimum, aspects of discrimination and calibration are extracted. For a full appraisal of models across multiple studies, systematic reviews should also record whether the primary study actually evaluated both calibration and discrimination. The absence of either component makes a full appraisal of prediction models difficult.

**Model evaluation.** When the predictive performance measures described above are evaluated or estimated in the same dataset used to develop the model, they are termed "apparent performance". The apparent performance tends to be biased (i.e., overestimated relative to performance in other individuals). Regardless of which modelling technique (regression, neural network, or machine learning techniques) is used, this risk of bias is more pronounced when the development dataset is small, the number of candidate predictors is large relative to the number of outcomes, data-driven predictor selection techniques have been applied, and shrinkage techniques have not been used. The assessment of the performance of prediction models should not rely on the development dataset, but rather be evaluated on other data. In fact, evaluation in an independent dataset is all that matters; how the model was derived is of minor importance [49]. Quantifying model performance in other individuals is often referred to as model validation (Box 1) [1,32,49,51,56,58,59, 119,129,130]. Several strategies exist depending on the availability of data, but are broadly categorised as internal and external validation [1,32,49,51,56,58,59,129].

Often the original dataset is randomly divided into a development sample and validation sample. However, this approach merely creates two similar but smaller datasets differing only by chance, and generally provides little additional information beyond the apparent performance, and for large datasets, the difference in performance in the development and validation dataset is even negligible, as expected [3,32,56,58,131]. Moreover, the method is statistically inefficient because not all available data are used to develop the prediction model, increasing the likelihood of overfitting, particularly in small datasets. Thus, for small datasets, the use of split-sample methods actually increases the risk of bias, whilst for large datasets there is no practical benefit [61]. If splitting the data is to be considered in large datasets, then a non-random split is preferable, for example splitting by time, centre, or geographic location [49,51,56,58,59,129].

Internal validation using resampling (Box 1) is a method to estimate the amount of overfitting or optimism in the apparent predictive performance of the developed model, for which no other data than the original study sample is used. Internal validation by resampling quantifies bias in the apparent model performance. Rather than cross validation, bootstrapping resampling methods are generally regarded as the preferred internal validation method, as all the data is used for model development and for model evaluation. Regardless of the modelling technique, bootstrapping is particularly recommended in small datasets with many candidate predictors and when predictor selection techniques have been used [3,49,51, 55,58,59]. In addition to capturing optimism in model performance, bootstrapping provides a shrinkage factor to adjust the estimated regression coefficients and apparent model performance for such overfitting.

Preferably, the predictive performance of a model is quantified in data that were not part of the development study data, but external to it (Type 3, Box 1). External data can differ in time (temporal validation) or location (geographical validation) from the data used to derive the prediction model. Usually this second dataset is comparable to the first, for example, in patients' clinical and demographic characteristics, reflecting the target population of the model development study. Sometimes, however, it is of interest to examine whether a model can also have predictive ability in other scenarios. For example, a validation dataset may differ in the clinical setting of participants (e.g., primary care versus secondary care), in the age range of participants (children versus adults), in the clinical inclusion criteria, or even by using different predictor or outcome definitions and measurements [1,31,32,56,129,132]. A crucial point is that a validation study should evaluate the exact published model (formula) derived from the initial data. Repeating the original modelling process in the validation data, refitting the model on new data, or fitting the linear predictor (in case a regression modelling technique was used) as a single term on the new data is not model validation, but rather model re-development [3,32,49,51,56,58,119]. If an existing model shows poor performance when evaluated in other individuals, researchers may adjust, update, or recalibrate the original model based on the validation data to increase performance. Such updating may range from adjusting the baseline risk (intercept or hazard) of the original model, to adjusting the predictor weights or regression coefficients, to adding new predictors or deleting existing predictors from the model [53,58,133]. Model updating, if done, usually follows an external validation of a previously published prediction model (Type 3, Box 1).

Systematic reviews should thus identify whether reported performance measures of the prediction models were obtained using only the development data (apparent performance), were corrected for optimism (e.g., using resampling techniques), used a random split-sample approach, or were based on performance in separate (external) datasets. If separate datasets have been used to develop and validate a prediction model, it is important to report any differences between the datasets. Updating or recalibrating a model based on external data should also be reported, if done. External validation studies provide the best insight into the performance of a model, indicating how useful it might be in other participants, centres, regions, or settings. However, many reviews have shown that external validation studies are generally uncommon [1,12,14,15,27,29,56,78].

**Results.** The results of the models in the review should match the systematic review question. If the aim is to review all existing prediction models in a particular clinical area, or for a particular outcome or groups of individuals (Box 2), results may include the components of the different models that have been developed, including the selected predictors, predictor weights, or regression coefficients (in case a regression approach was used) and their precision estimates, in addition to the performance of these models [12,27,29,45]. If the aim is to review the reproducibility or predictive performance of the same model(s) across different study samples (external validation), as for example in [44,134,139], the predictive accuracy measures and their precision estimates are important to focus on, whilst issues surrounding the development of the models are less relevant to report.

As models are usually developed to estimate an individual's outcome probability, it is important to capture and record whether this can actually be done from the published model. The format used to present models in the original papers should be extracted. Options include the original model formula (e.g., the regression equation if a regression approach was used to develop the model) enabling direct probability estimation, rounded scoring rules, or predefined risk groups with corresponding predicted and observed outcome probabilities. Rounding or simplifying original predictor weights or regression coefficients is likely to cause a loss in predictive accuracy. Hence, if relevant, the systematic review should report the performance measures of the original and "rounded" models where this information is available in the published primary report [32].

Risk groups are frequently presented. For reasons described above, data driven methods to create risk groups, such as the "optimal" probability threshold method or at the median, are not recommended [135]. Therefore, it is important to note if and how risk groups were created. Recent reviews in oncology highlighted poor methods and poor reporting for creating risk groups [56,60].

When a review includes both development and validation studies of the same model(s), or several external validations of the same model, reporting differences in frequency (binary) and distribution (continuous) of the predictors and outcomes across the study samples is recommended, as a different case-mix is known to result in different predicted risks that may influence model performance measures [49,53,129,133,136,137].

**Interpretation and discussion.** All tools for reporting of medical studies recommend discussing strengths, weaknesses, and future challenges of a study and its reported results [33–37], including the PRISMA statement for reporting of systematic reviews itself [38]. How a model was developed and validated and its reported performance give insight into whether the reviewed model is likely to be useful, and for whom. Conclusions about model performance and applicability should be based on the validation results of the model, the comparison with other studies and other prediction models, and study strengths and weaknesses,

rather than predictor effects or corresponding p-values. Furthermore, one may like to overview the performance of all prediction models for a specific outcome or target population before making decisions on which model to apply in routine practice [138].

## Conclusion

In contrast to systematic reviews of therapeutic and diagnostic test accuracy studies, there is no formal checklist for guidance on defining a proper review question, let alone for data extraction or critical appraisal of primary studies on the development or validation of diagnostic or prognostic prediction models, despite the sharp increase of such studies in the past decade. We combined published risk-of-bias tools, existing critical appraisal checklists for systematic reviews of randomised therapeutic trials and diagnostic test accuracy research, methodological recommendations for conduct and reporting of prediction model research, and data extraction sheets used in published reviews of prediction modelling studies to provide the CHARMS checklist. The checklist is intended to help frame the review question, design the review, and extract the relevant items from the reports of the primary prediction modelling studies and to guide assessment of the risk of bias and the applicability of the reviewed prediction models. We recognise that this checklist will require further evaluation and use to adjust and improve CHARMS.

## Supporting Information

**Text S1** A one-page checklist of relevant items to extract from individual studies in a systematic review of prediction models. (DOCX)

## Author Contributions

Wrote the first draft of the manuscript: KGMM JAHdG GSC. Wrote the paper: KGMM JAHdG WB YV SM DGA JBR GSC. ICMJE criteria for authorship read and met: KGMM JAHdG WB YV SM DGA JBR GSC. Agree with manuscript results and conclusions: KGMM JAHdG WB YV SM DGA JBR GSC.

## References

1. Reilly BM, Evans AT (2006) Translating clinical research into clinical practice: impact of using prediction rules to make decisions. Ann Intern Med 144: 201–209.
2. Bouwmeester W, Zuithoff NP, Mallett S, Geerlings MI, Vergouwe Y, et al. (2012) Reporting and methods in clinical prediction research: a systematic review. PLoS Med 9: 1–12.
3. Steyerberg EW, Moons KG, van der Windt DA, Hayden JA, Perel P, et al. (2013) Prognosis Research Strategy (PROGRESS) 3: prognostic model research. PLoS Med 10: e1001381.
4. Wells PS, Hirsh J, Anderson DR, Lensing AW, Foster G, et al. (1998) A simple clinical model for the diagnosis of deep-vein thrombosis combined with impedance plethysmography: potential for an improvement in the diagnostic process. J Intern Med 243: 15–23.
5. Oudega R, Moons KG, Hoes AW (2005) Ruling out deep venous thrombosis in primary care. A simple diagnostic algorithm including D-dimer testing. Thromb Haemost 94: 200–205.
6. Stiell IG, Greenberg GH, McKnight RD, Nair RC, McDowell I, et al. (1993) Decision rules for the use of radiography in acute ankle injuries. Refinement and prospective validation. JAMA 269: 1127–1132.
7. Rietveld RP, ter Riet G, Bindels PJ, Sloos JH, van Weert HC (2004) Predicting bacterial cause in infectious conjunctivitis: cohort study on informativeness of combinations of signs and symptoms. BMJ 329: 206–210.
8. Galea MH, Blamey RW, Elston CE, Ellis IO (1992) The Nottingham Prognostic Index in primary breast cancer. Breast Cancer Res Treat 22: 207–219.
9. Nashef SA, Roques F, Michel P, Gauducheau E, Lemeshow S, et al. (1999) European system for cardiac operative risk evaluation (EuroSCORE). Eur J Cardiothorac Surg 16: 9–13.
10. Wilson PW, D'Agostino RB, Levy D, Belanger AM, Silbershatz H, et al. (1998) Prediction of coronary heart disease using risk factor categories. Circulation 97: 1837–1847.
11. Lindstrom J, Tuomilehto J (2003) The diabetes risk score: a practical tool to predict type 2 diabetes risk. Diabetes Care 26: 725–731.
12. Perel P, Edwards P, Wentz R, Roberts I (2006) Systematic review of prognostic models in traumatic brain injury. BMC Med Inform Decis Mak 6: 38.
13. Altman (2007) Prognostic models: a methodological framework and review of models for breast cancer. In: Lyman GH, Burstein HJ, editor. Breast cancer Translational therapeutic strategies. New York Informa Healtcare. pp. 11–25.
14. van Dieren S, Beulens JW, Kengne AP, Peelen LM, Rutten GE, et al. (2012) Prediction models for the risk of cardiovascular disease in patients with type 2 diabetes: a systematic review. Heart 98: 360–369.
15. Collins GS, Mallett S, Omar O, Yu LM (2011) Developing risk prediction models for type 2 diabetes: a systematic review of methodology and reporting. BMC Med 9: 103.
16. Ettema RG, Peelen LM, Schuurmans MJ, Nierich AP, Kalkman CJ, et al. (2010) Prediction models for prolonged intensive care unit stay after cardiac surgery: systematic review and validation study. Circulation 122: 682–689.
17. Rabar S, Lau R, O'Flynn N, Li L, Barry P (2012) Risk assessment of fragility fractures: summary of NICE guidance. BMJ 345: e3698.
18. Goff DC Jr, Lloyd-Jones DM, Bennett G, Coady S, D'Agostino RB Sr, et al. (2013) 2013 ACC/AHA Guideline on the Assessment of Cardiovascular Risk: A Report of the American College of Cardiology/American Heart Association Task Force on Practice Guidelines. Circulation 129: S49–S73.
19. Riley RD, Ridley G, Williams K, Altman DG, Hayden J, et al. (2007) Prognosis research: toward evidence-based results and a Cochrane methods group. J Clin Epidemiol 60: 863–865; author reply 865–866.
20. Hemingway H (2006) Prognosis research: why is Dr. Lydgate still waiting? J Clin Epidemiol 59: 1229–1238.
21. Ingui BJ, Rogers MA (2001) Searching for clinical prediction rules in MEDLINE. J Am Med Inform Assoc 8: 391–397.
22. Wong SS, Wilczynski NL, Haynes RB, Ramkissoonsingh R, Hedges Team (2003) Developing optimal search strategies for detecting sound clinical prediction studies in MEDLINE. AMIA Annu Symp Proc 2003: 728–732.
23. Keogh C, Wallace E, O'Brien KK, Murphy PJ, Teljeur C, et al. (2011) Optimized retrieval of primary care clinical prediction rules from MEDLINE to establish a Web-based register. J Clin Epidemiol 64: 848–860.
24. Geersing GJ, Bouwmeester W, Zuithoff P, Spijker R, Leeflang M, et al. (2012) Search filters for finding prognostic and diagnostic prediction studies in Medline to enhance systematic reviews. PLoS ONE 7: e32844.
25. Hayden JA, Cote P, Bombardier C (2006) Evaluation of the quality of prognosis studies in systematic reviews. Ann Intern Med 144: 427–437.
26. Hayden JA, van der Windt DA, Cartwright JL, Cote P, Bombardier C (2013) Assessing bias in studies of prognostic factors. Ann Intern Med 158: 280–286.
27. Counsell C, Dennis M (2001) Systematic review of prognostic models in patients with acute stroke. Cerebrovasc Dis 12: 159–170.
28. Tamariz LJ, Eng J, Segal JB, Krishnan JA, Bolger DT, et al. (2004) Usefulness of clinical prediction rules for the diagnosis of venous thromboembolism: A systematic review. Am J Med 117: 676.
29. Leushuis E, van der Steeg JW, Steures P, Bossuyt PM, Eijkemans MJ, et al. (2009) Prediction models in reproductive medicine: a critical appraisal. Hum Reprod Update 15: 537–552.
30. Mallett S, Timmer A, Sauerbrei W, Altman DG (2010) Reporting of prognostic studies of tumour markers: a review of published articles in relation to REMARK guidelines. Br J Cancer 102: 173–180.
31. Moons KG, Altman DG, Vergouwe Y, Royston P (2009) Prognosis and prognostic research: application and impact of prognostic models in clinical practice. BMJ 338: 1487–1490.
32. Moons KG, Kengne AP, Grobbee DE, Royston P, Vergouwe Y, et al. (2012) Risk prediction models: II. External validation, model updating, and impact assessment. Heart 98: 691–698.
33. Moher D, Hopewell S, Schulz KF, Montori V, Gotzsche PC, et al. (2010) CONSORT 2010 Explanation and Elaboration: Updated guidelines for reporting parallel group randomised trials. J Clin Epidemiol 63: e1–37.
34. McShane LM, Altman DG, Sauerbrei W, Taube SE, Gion M, et al. (2005) REporting recommendations for tumour MARKer prognostic studies (REMARK). Br J Cancer 93: 387–391.
35. Bossuyt PM, Reitsma JB, Bruns DE, Gatsonis CA, Glasziou PP, et al. (2003) The STARD statement for reporting studies of diagnostic accuracy: explanation and elaboration. Clin Chem 49: 7–18.
36. von Elm E, Altman DG, Egger M, Pocock SJ, Gotzsche PC, et al. (2007) The Strengthening the Reporting of Observational Studies in Epidemiology (STROBE) statement: guidelines for reporting observational studies. Ann Intern Med 147: 573–577.
37. Janssens AC, Ioannidis JP, Bedrosian S, Boffetta P, Dolan SM, et al. (2011) Strengthening the reporting of genetic risk prediction studies (GRIPS): explanation and elaboration. Eur J Epidemiol 26: 313–337.
38. Liberati A, Altman DG, Tetzlaff J, Mulrow C, Gotzsche PC, et al. (2009) The PRISMA Statement for Reporting Systematic Reviews and Meta-Analyses of Studies That Evaluate Health Care Interventions: Explanation and Elaboration. Ann Intern Med 151: W65–W94.

39. Higgins JP, Altman DG, Gotzsche PC, Juni P, Moher D, et al. (2011) The Cochrane Collaboration's tool for assessing risk of bias in randomised trials. BMJ 343: d5928.
40. Whiting P, Rutjes AW, Reitsma JB, Bossuyt PM, Kleijnen J (2003) The development of QUADAS: a tool for the quality assessment of studies of diagnostic accuracy included in systematic reviews. BMC Med Res Methodol 3: 25.
41. Whiting PF, Rutjes AW, Westwood ME, Mallett S, Deeks JJ, et al. (2011) QUADAS-2: a revised tool for the quality assessment of diagnostic accuracy studies. Ann Intern Med 155: 529–536.
42. Altman DG (2001) Systematic reviews of evaluations of prognostic variables. BMJ 323: 224–228.
43. Kyzas PA, Denaxa-Kyza D, Ioannidis JP (2007) Quality of reporting of cancer prognostic marker studies: association with reported prognostic effect. J Natl Cancer Inst 99: 236–243.
44. Siontis GC, Tzoulaki I, Siontis KC, Ioannidis JP (2012) Comparisons of established risk prediction models for cardiovascular disease: systematic review. BMJ 344: e3318.
45. Shariat SF, Karakiewicz PI, Margulis V, Kattan MW (2008) Inventory of prostate cancer predictive tools. Curr Opin Urol 18: 279–296.
46. Veerbeek JM, Kwakkel G, van Wegen EE, Ket JC, Heymans MW (2011) Early prediction of outcome of activities of daily living after stroke: a systematic review. Stroke 42: 1482–1488.
47. Moons KG, Royston P, Vergouwe Y, Grobbee DE, Altman DG (2009) Prognosis and prognostic research: what, why, and how? BMJ 338: 1317–1320.
48. Laupacis A, Sekar N, Stiell IG (1997) Clinical prediction rules. A review and suggested modifications of methodological standards. JAMA 277: 488–494.
49. Altman DG, Royston P (2000) What do we mean by validating a prognostic model? Stat Med 19: 453–473.
50. McGinn TG, Guyatt GH, Wyer PC, Naylor CD, Stiell IG, et al. (2000) Users' guides to the medical literature: XXII: how to use articles about clinical decision rules. Evidence-Based Medicine Working Group. JAMA 284: 79–84.
51. Harrell FE (2001) Regression Modeling Strategies. New York: Springer-Verlag.
52. Moons KG, Grobbee DE (2002) Diagnostic studies as multivariable, prediction research. J Epidemiol Community Health 56: 337–338.
53. Janssen KJ, Moons KG, Kalkman CJ, Grobbee DE, Vergouwe Y (2008) Updating methods improved the performance of a clinical prediction model in new patients. J Clin Epidemiol 61: 76–86.
54. Grobbee DE, Hoes AW (2009) Clinical Epidemiology - Principles, Methods and Applications for Clinical Research. London: Jones and Bartlett Publishers. 413 pp.
55. Royston P, Moons KG, Altman DG, Vergouwe Y (2009) Prognosis and prognostic research: Developing a prognostic model. BMJ 338: b604.
56. Altman DG, Vergouwe Y, Royston P, Moons KG (2009) Prognosis and prognostic research: validating a prognostic model. BMJ 338: 1432–1435.
57. McGeechan K, Macaskill P, Irwig L, Liew G, Wong TY (2008) Assessing new biomarkers and predictive models for use in clinical practice: a clinician's guide. Arch Intern Med 168: 2304–2310.
58. Steyerberg EW (2009) Clinical prediction models: A practical approach to development, validation, and updating; Gail M, Krickeberg K, Samet J, Tsiati A, Wong W, editors. Rotterdam: Springer. 497 p.
59. Moons KG, Kengne AP, Woodward M, Royston P, Vergouwe Y, et al. (2012) Risk prediction models: I. Development, internal validation, and assessing the incremental value of a new (bio)marker. Heart 98: 683–690.
60. Mallett S, Royston P, Dutton S, Waters R, Altman DG (2010) Reporting methods in studies developing prognostic models in cancer: a review. BMC Med 8: 20.
61. Jacob M, Bruegger D, Conzen P, Becker BF, Finsterer U, et al. (2005) Development and validation of a mathematical algorithm for quantifying preoperative blood volume by means of the decrease in hematocrit resulting from acute normovolemic hemodilution. Transfusion 45: 562–571.
62. Collins GS, Omar O, Shanyinde M, Yu LM (2013) A systematic review finds prediction models for chronic kidney disease were poorly reported and often developed using inappropriate methods. J Clin Epidemiol 66: 268–277.
63. Vickers AJ, Cronin AM (2010) Everything you always wanted to know about evaluating prediction models (but were too afraid to ask). Urology 76: 1298–1301.
64. Bolland MJ, Jackson R, Gamble GD, Grey A (2013) Discrepancies in predicted fracture risk in elderly people. BMJ 346: e8669.
65. Ganna A, Reilly M, de Faire U, Pedersen N, Magnusson P, et al. (2012) Risk prediction measures for case-cohort and nested case-control designs: an application to cardiovascular disease. Am J Epidemiol 175: 715–724.
66. Biesheuvel CJ, Vergouwe Y, Oudega R, Hoes AW, Grobbee DE, et al. (2008) Advantages of the nested case-control design in diagnostic research. BMC Med Res Methodol 8: 48.
67. Rutjes AW, Reitsma JB, Vandenbroucke JP, Glas AS, Bossuyt PM (2005) Case-control and two-gate designs in diagnostic accuracy studies. Clin Chem 51: 1335–1341.
68. van Zaane B, Vergouwe Y, Donders AR, Moons KG (2012) Comparison of approaches to estimate confidence intervals of post-test probabilities of diagnostic test results in a nested case-control study. BMC Med Res Methodol 12: 166.
69. Oostenbrink R, Moons KG, Bleeker SE, Moll HA, Grobbee DE (2003) Diagnostic research on routine care data: prospects and problems. J Clin Epidemiol 56: 501–506.
70. Geomini P, Kruitwagen R, Bremer GL, Cnossen J, Mol BW (2009) The accuracy of risk scores in predicting ovarian malignancy: a systematic review. Obstet Gynecol 113: 384–394.
71. Stiell IG, Wells GA (1999) Methodologic standards for the development of clinical decision rules in emergency medicine. Ann Emerg Med 33: 437–447.
72. Altman DG, Schulz KF, Moher D, Egger M, Davidoff F, et al. (2001) The revised CONSORT statement for reporting randomized trials: explanation and elaboration. Ann Intern Med 134: 663–694.
73. Knottnerus JA (2002) Challenges in dia-prognostic research. J Epidemiol Community Health 56: 340–341.
74. Oudega R, Moons KG, Hoes AW (2005) Limited value of patient history and physical examination in diagnosing deep vein thrombosis in primary care. Fam Pract 22: 86–91.
75. Beneciuk JM, Bishop MD, George SZ (2009) Clinical prediction rules for physical therapy interventions: a systematic review. Phys Ther 89: 114–124.
76. Minne L, Eslami S, de Keizer N, de Jonge E, de Rooij SE, et al. (2012) Effect of changes over time in the performance of a customized SAPS-II model on the quality of care assessment. Intensive Care Med 38: 40–46.
77. Collins GS, Altman DG (2012) Predicting the 10 year risk of cardiovascular disease in the United Kingdom: independent and external validation of an updated version of QRISK2. BMJ 344: e4181.
78. Mallett S, Royston P, Waters R, Dutton S, Altman DG (2010) Reporting performance of prognostic models in cancer: a review. BMC Med 8: 21.
79. Rutjes AW, Reitsma JB, Di Nisio M, Smidt N, van Rijn JC, et al. (2006) Evidence of bias and variation in diagnostic accuracy studies. CMAJ 174: 469–476.
80. Whiting P, Rutjes AW, Reitsma JB, Glas AS, Bossuyt PM, et al. (2004) Sources of variation and bias in studies of diagnostic accuracy: a systematic review. Ann Intern Med 140: 189–202.
81. Hess EP, Thiruganasambandamoorthy V, Wells GA, Erwin P, Jaffe AS, et al. (2008) Diagnostic accuracy of clinical prediction rules to exclude acute coronary syndrome in the emergency department setting: a systematic review. CJEM 10: 373–382.
82. Ferreira-Gonzalez I, Busse JW, Heels-Ansdell D, Montori VM, Akl EA, et al. (2007) Problems with use of composite end points in cardiovascular trials: systematic review of randomised controlled trials. Bmj 334: 786.
83. Glynn RJ, Rosner B (2004) Methods to evaluate risks for composite end points and their individual components. J Clin Epidemiol 57: 113–122.
84. Gondrie MJ, Janssen KJ, Moons KG, van der Graaf Y (2012) A simple adaptation method improved the interpretability of prediction models for composite end points. J Clin Epidemiol 65: 946–953.
85. Lijmer JG, Mol BW, Heisterkamp S, Bonsel GJ, Prins MH, et al. (1999) Empirical evidence of design-related bias in studies of diagnostic tests. JAMA 282: 1061–1066.
86. Maguire JL, Boutis K, Uleryk EM, Laupacis A, Parkin PC (2009) Should a head-injured child receive a head CT scan? A systematic review of clinical prediction rules. Pediatrics 124: e145–154.
87. Serrano LA, Hess EP, Bellolio MF, Murad MH, Montori VM, et al. (2010) Accuracy and quality of clinical decision rules for syncope in the emergency department: a systematic review and meta-analysis. Ann Emerg Med 56: 362–373 e361.
88. Reitsma JB, Rutjes AW, Khan KS, Coomarasamy A, Bossuyt PM (2009) A review of solutions for diagnostic accuracy studies with an imperfect or missing reference standard. J Clin Epidemiol 62: 797–806.
89. Rutjes A, Reitsma J, Coomarasamy A, Khan K, Bossuyt P (2007) Evaluation of diagnostic tests when there is no gold standard. A review of methods. Health Technol Assess 50: ix-51.
90. Walraven vC, Davis D, Forster AJ, Wells GA (2004) Time-dependent bias was common in survival analyses published in leading clinical journals. J Clin Epidemiol 57: 672–682.
91. Moons KG, Grobbee DE (2002) When should we remain blind and when should our eyes remain open in diagnostic studies? J Clin Epidemiol 55: 633–636.
92. Bennette C, Vickers A (2012) Against quantiles: categorization of continuous variables in epidemiologic research, and its discontents. BMC Med Res Methodol 12: 21.
93. Royston P, Altman DG, Sauerbrei W (2006) Dichotomizing continuous predictors in multiple regression: a bad idea. Stat Med 25: 127–141.
94. Altman DG, Royston P (2006) The cost of dichotomising continuous variables. BMJ 332: 1080.
95. Leeflang MM, Moons KG, Reitsma JB, Zwinderman AH (2008) Bias in sensitivity and specificity caused by data-driven selection of optimal cutoff values: mechanisms, magnitude, and solutions. Clin Chem 54: 729–737.
96. Royston P, Sauerbrei W, Altman DG (2000) Modeling the effects of continuous risk factors. J Clin Epidemiol 53: 219–221.
97. Concato J, Peduzzi P, Holford TR, Feinstein AR (1995) Importance of events per independent variable in proportional hazards analysis. I. Background, goals, and general strategy. J Clin Epidemiol 48: 1495–1501.
98. Peduzzi P, Concato J, Feinstein AR, Holford TR (1995) Importance of events per independent variable in proportional hazards regression analysis. II.

Accuracy and precision of regression estimates. J Clin Epidemiol 48: 1503–1510.
99. Peduzzi P, Concato J, Kemper E, Holford TR, Feinstein AR (1996) A simulation study of the number of events per variable in logistic regression analysis. J Clin Epidemiol 49: 1373–1379.
100. Vittinghoff E, McCulloch CE (2007) Relaxing the rule of ten events per variable in logistic and Cox regression. Am J Epidemiol 165: 710–718.
101. Courvoisier DS, Combescure C, Agoritsas T, Gayet-Ageron A, Perneger TV (2011) Performance of logistic regression modeling: beyond the number of events per variable, the role of data structure. J Clin Epidemiol 64: 993–1000.
102. Vergouwe Y, Steyerberg EW, Eijkemans MJ, Habbema JD (2005) Substantial effective sample sizes were required for external validation studies of predictive logistic regression models. J Clin Epidemiol 58: 475–483.
103. Burton A, Altman DG (2004) Missing covariate data within cancer prognostic studies: a review of current reporting and proposed guidelines. Br J Cancer 91: 4–8.
104. Mackinnon A (2010) The use and reporting of multiple imputation in medical research - a review. J Intern Med 268: 586–593.
105. Little RJA (1992) Regression with missing X's: A review. JASA 87: 1227–1237.
106. Moons KG, Donders RA, Stijnen T, Harrell FE Jr (2006) Using the outcome for imputation of missing predictor values was preferred. J Clin Epidemiol 59: 1092–1101.
107. Gorelick MH (2006) Bias arising from missing data in predictive models. J Clin Epidemiol 59: 1115–1123.
108. Wood AM, White IR, Royston P (2008) How should variable selection be performed with multiply imputed data? Stat Med 27: 3227–3246.
109. Janssen KJ, Donders AR, Harrell FE Jr, Vergouwe Y, Chen Q, et al. (2010) Missing covariate data in medical research: to impute is better than to ignore. J Clin Epidemiol 63: 721–727.
110. Janssen KJ, Vergouwe Y, Donders AR, Harrell FE Jr, Chen Q, et al. (2009) Dealing with missing predictor values when applying clinical prediction models. Clin Chem 55: 994–1001.
111. Vergouwe Y, Royston P, Moons KG, Altman DG (2010) Development and validation of a prediction model with missing predictor data: a practical approach. J Clin Epidemiol 63: 205–214.
112. Marshall A, Altman DG, Royston P, Holder RL (2010) Comparison of techniques for handling missing covariate data within prognostic modelling studies: a simulation study. BMC Med Res Methodol 10: 7.
113. Donders AR, van der Heijden GJ, Stijnen T, Moons KG (2006) Review: a gentle introduction to imputation of missing values. J Clin Epidemiol 59: 1087–1091.
114. Sterne JA, White IR, Carlin JB, Spratt M, Royston P, et al. (2009) Multiple imputation for missing data in epidemiological and clinical research: potential and pitfalls. BMJ 338: b2393.
115. White IR, Royston P, Wood AM (2011) Multiple imputation using chained equations: Issues and guidance for practice. Stat Med 30: 377–399.
116. Sun GW, Shook TL, Kay GL (1996) Inappropriate use of bivariable analysis to screen risk factors for use in multivariable analysis. J Clin Epidemiol 49: 907–916.
117. Harrell FE, Lee KL, Mark DB (1996) Multivariable prognostic models: issues in developing models, evaluating assumptions and adequacy, and measuring and reducing erros. Stat Med 15: 361–387.
118. Houwelingen van JC, Le Cessie S (1990) Predictive value of statistical models. Stat Med 9: 1303–1325.
119. Royston P, Altman DG (2013) External validation of a cox prognostic model: principles and methods. BMC Med Res Methodol 13: 33.
120. Steyerberg EW, Eijkemans MJ, Harrell FE, Habbema JD (2001) Prognostic modeling with logistic regression analysis: in search of a sensible strategy in small data sets. Med Decis Making 21: 45–56.
121. Peek N, Arts DG, Bosman RJ, van der Voort PH, de Keizer NF (2007) External validation of prognostic models for critically ill patients required substantial sample sizes. J Clin Epidemiol 60: 491–501.
122. Steyerberg EW, Vickers AJ, Cook NR, Gerds T, Gonen M, et al. (2010) Assessing the performance of prediction models: a framework for traditional and novel measures. Epidemiology 21: 128–138.
123. Cook NR (2008) Statistical evaluation of prognostic versus diagnostic models: beyond the ROC curve. Clin Chem 54: 17–23.
124. Pencina MJ, D'Agostino RB Sr, D'Agostino RB Jr, Vasan RS (2008) Evaluating the added predictive ability of a new marker: from area under the ROC curve to reclassification and beyond. Stat Med 27: 157–172; discussion 207–112.
125. Pepe MS, Feng Z, Gu JW (2008) Comments on 'Evaluating the added predictive ability of a new marker: From area under the ROC curve to reclassification and beyond' by M. J. Pencina et al., Statistics in Medicine (DOI: 10.1002/sim.2929). Stat Med 27: 173–181.
126. Pencina MJ, D'Agostino RB Sr, Steyerberg EW (2011) Extensions of net reclassification improvement calculations to measure usefulness of new biomarkers. Stat Med 30: 11–21.
127. Pepe MS (2011) Problems with risk reclassification methods for evaluating prediction models. Am J Epidemiol 173: 1327–1335.
128. Vickers AJ, Elkin EB (2006) Decision curve analysis: a novel method for evaluating prediction models. Med Decis Making 26: 565–574.
129. Justice AC, Covinsky KE, Berlin JA (1999) Assessing the generalizability of prognostic information. Ann Intern Med 130: 515–524.
130. Vergouwe Y, Moons KG, Steyerberg EW (2010) External validity of risk models: Use of benchmark values to disentangle a case-mix effect from incorrect coefficients. Am J Epidemiol 172: 971–980.
131. Steyerberg EW, Eijkemans MJ, Harrell FE Jr, Habbema JD (2000) Prognostic modelling with logistic regression analysis: a comparison of selection and estimation methods in small data sets. Stat Med 19: 1059–1079.
132. Toll DB, Janssen KJ, Vergouwe Y, Moons KG (2008) Validation, updating and impact of clinical prediction rules: a review. J Clin Epidemiol 61: 1085–1094.
133. Houwelingen van JC (2000) Validation, calibration, revision and combination of prognostic survival models. Stat Med 19: 3401–3415.
134. Tzoulaki I, Liberopoulos G, Ioannidis JP (2009) Assessment of claims of improved prediction beyond the Framingham risk score. JAMA 302: 2345–2352.
135. Altman DG, Lyman GH (1998) Methodological challenges in the evaluation of prognostic factors in breast cancer. Breast Cancer Res Treat 52: 289–303.
136. Vergouwe Y, Steyerberg EW, de Wit R, Roberts JT, Keizer HJ, et al. (2003) External validity of a prediction rule for residual mass histology in testicular cancer: an evaluation for good prognosis patients. Br J Cancer 88: 843–847.
137. Hukkelhoven CW, Rampen AJ, Maas AI, Farace E, Habbema JD, et al. (2006) Some prognostic models for traumatic brain injury were not valid. J Clin Epidemiol 59: 132–143.
138. Collins GS, Moons KG (2012) Comparing risk prediction models. BMJ 344: e3186.
139. Siregar S, Groenwold RH, de Heer F, Bots ML, van der Graaf Y, et al. (2012) Performance of the original EuroSCORE. Eur J Cardiothorac Surg 41: 746–754.
140. Peters SA, den Ruijter HM, Bots ML, Moons KG (2012) Improvements in risk stratification for the occurrence of cardiovascular disease by imaging subclinical atherosclerosis: a systematic review. Heart 98: 177–184.
